# A Systematic Framework for Prioritizing Burden of Disease Data Required for Vaccine Development and Implementation: The Case for Group A Streptococcal Diseases^[Author-notes ciac291-FM3]^

**DOI:** 10.1093/cid/ciac291

**Published:** 2022-04-19

**Authors:** Hannah C Moore, Jeffrey W Cannon, David C Kaslow, Theresa Lamagni, Asha C Bowen, Kate M Miller, Thomas Cherian, Jonathan Carapetis, Chris Van Beneden

**Affiliations:** Wesfarmers Centre for Vaccines and Infectious Diseases, Telethon Kids Institute, University of Western Australia, Perth, Western Australia, Australia; Wesfarmers Centre for Vaccines and Infectious Diseases, Telethon Kids Institute, University of Western Australia, Perth, Western Australia, Australia; Department of Global Health and Population, Harvard T.H. Chan School of Public Health, Boston, Massachusetts, USA; PATH, Seattle, Washington, USA; UK Health Security Agency, London, United Kingdom; Wesfarmers Centre for Vaccines and Infectious Diseases, Telethon Kids Institute, University of Western Australia, Perth, Western Australia, Australia; Perth Children’s Hospital, Perth, Western Australia, Australia; Wesfarmers Centre for Vaccines and Infectious Diseases, Telethon Kids Institute, University of Western Australia, Perth, Western Australia, Australia; MMGH Consulting, Geneva, Switzerland; Wesfarmers Centre for Vaccines and Infectious Diseases, Telethon Kids Institute, University of Western Australia, Perth, Western Australia, Australia; Perth Children’s Hospital, Perth, Western Australia, Australia; CDC Foundation, Atlanta, Georgia, USA

**Keywords:** burden of disease, vaccine policy, vaccine development, group A streptococcal diseases

## Abstract

Vaccine development and implementation decisions need to be guided by accurate and robust burden of disease data. We developed an innovative systematic framework outlining the properties of such data that are needed to advance vaccine development and evaluation, and prioritize research and surveillance activities. We focus on 4 objectives—advocacy, regulatory oversight and licensure, policy and post-licensure evaluation, and post-licensure financing—and identify key stakeholders and specific requirements for burden of disease data aligned with each objective. We apply this framework to group A *Streptococcus,* a pathogen with an underrecognized global burden, and give specific examples pertinent to 8 clinical endpoints. This dynamic framework can be adapted for any disease with a vaccine in development and can be updated as vaccine candidates progress through clinical trials. This framework will also help with research and innovation priority setting of the Immunization Agenda 2030 (IA2030) and accelerate development of future vaccines.

## Teaser

We introduce an innovative systematic framework prioritizing burden of disease data requirements for vaccines through advocacy, regulatory and licensing, policy and post-introduction evaluation, and financing. We apply this framework to group A *Streptococcus* to aid efforts to accelerate vaccine development.

Vaccines are an important public health tool to prevent infectious diseases and improve population health and well-being [1, 2]. Multiple factors influence decision making during vaccine development, from the preclinical phase through large-scale vaccine trials and policy formation. Establishing credible burden of disease estimates should be the cornerstone of new vaccine development [3]; however, decisions are not always informed by such estimates. “Burden of disease” encompasses a broad range of outcomes, including the entire spectrum of associated morbidities and sequelae, and health-related costs of an illness. It incorporates multiple data types, including acute disease incidence, disability-adjusted life-years (DALYs), health service utilization rates, prevalence of associated chronic diseases, and mortality rates. The perceived credibility of the estimates and how they are packaged and presented within the context of overall public health priorities can influence vaccine development and implementation. Thus, the importance of accurate, robust data should neither be dismissed nor underestimated. High-quality data can be used in post-implementation evaluations to measure and improve vaccine program performance. Furthermore, data-enabled decision making is 1 of 4 core principles in the World Health Organization’s (WHO’s) Immunization Agenda 2030 (IA2030) [4].

Beyond incorporating traditional disease burden measures, a systematic framework for prioritizing burden of disease should also include estimates of social and economic impact, community acceptability, and recognition of the need for adding vaccines to existing prevention and control measures. The global effort to rapidly develop, evaluate, and implement coronavirus disease 2019 (COVID-19) vaccines in the face of the largest global pandemic in modern history exemplifies how high rates of disease burden, widespread economic disruption, and the scientific, political, and community demand for vaccination can drive vaccine development [5]. This can potentially alter perceptions on the value of vaccines in preventing infectious diseases [6].

Despite the global success of some vaccines, vaccine development for other pathogens with an established high burden have faced impediments. Group A *Streptococcus* (*Streptococcus pyogenes*, herein referred to as Strep A), which is recognized as a WHO vaccination priority, is 1 example [7, 8]. Vaccine research and development investments for this pathogen remain minimal [9]. Despite an estimated annual 800 million infections and 639 000 deaths globally [10], the burden of Strep A disease remains underappreciated, especially in low- to middle-income countries (LMICs). There is a need to improve the robustness of Strep A disease burden estimates and widely communicate the potential value of vaccination to bolster the rationale for prioritizing vaccine development and implementation. Such a robust approach is a priority of the Wellcome Trust–funded Strep A Vaccine Consortium (SAVAC; www.savac.ivi.int).

We describe the properties of burden of disease data that are needed to progress vaccine development, evaluation, and policy making and a framework to prioritize studies and surveillance activities that would yield robust data addressing different vaccine objectives. Using this framework, we aimed to provide specific examples for Strep A and identify future research priorities.

## FRAMEWORK DEVELOPMENT

SAVAC established an expert Burden of Disease Working Group (BoDWG) comprising 13 members from 7 geographically diverse countries, with wide-ranging expertise in Strep A and other vaccine-preventable diseases (VPDs), disease surveillance, and vaccine implementation. The BoDWG identified and reached consensus on 4 overarching vaccine objectives (advocacy, regulatory oversight and licensure, policy and post-licensure evaluation, and post-licensure financing) through iterative discussion to build a framework (Table 1). We describe 4 key elements for consideration across each objective. For key stakeholders, the framework prioritizes the most relevant disease burden data necessary to inform vaccine clinical development and introduction activities. We illustrate the application of these vaccine objectives to existing vaccines (Supplementary Table 1).

**Table 1.** Framework for Prioritizing Burden of Disease Data for Vaccine Development and Evaluation Objectives

	Vaccine Objective			
Element	Advocacy	Regulatory/Licensure	Policy and Post-Licensure Evaluation	Financing
Stage of vaccine pipeline	All stages	Pre-licensure and licensure/pre-qualification stages, with some continuation for post-licensure commitments	Post-licensure, but early analyses needed pre-licensure period	Required for post-licensure decision making, but evidence needed pre-licensure for 5-year Vaccine Investment Strategy decision making by Gavi and others
Key audience/stakeholders	Public and private donors and funding bodies Public figures (eg, politicians and specialist physicians) and advocacy groups, especially in countries with high disease burden Manufacturers/developers (pharmaceutical and biotech companies) Wider community/society (eg, CSOs)	National government/regulators (NRAs) WHO vaccines pre-qualification Manufacturers/developers (pharmaceutical and biotech companies, public–private partnerships) Funders and donors	Global, regional, and national policy makers and advisors (eg, WHO, SAGE, GNN, RITAGs, NITAGs) Public sector immunization programs (eg, EPI Managers) In-country champions (eg, specialist physicians)	Multilateral funders (ie, Gavi and its Vaccine Investment Strategy) National government bodies (NITAGs, Ministries of Health and Finance) Industry/manufacturers Bilateral public and private funders Private medical insurance organizations
Data purpose	Quantify overall preventable burden of disease that are comparable across countries/regions Focus on data most likely to influence decision making (including individual vaccinees [and their caregivers]), of greatest public health significance Contextualize in relation to global, regional. or national public health and development goals (eg, SDGs, IA2030)	Provide foundation needed to design and plan clinical trials to measure vaccine efficacy and safety for key disease endpoints	Measure effectiveness post-licensure (which includes knowledge of disease epidemiology prior to vaccine introduction) Model and predict potential impact pre-licensure Provide evidence to form recommendations	Assess return on investment decisions
Overarching data requirements	Full disease spectrum Specific and nonspecific disease endpoints	Age-specific incidence of specific clinical endpoints as guided by WHO-preferred product characteristics in well-characterized populations	Vaccine-preventable disease burden (population-based, where feasible) Specific and nonspecific disease endpoints	Cost of vaccination to prevent disease (where feasible) Cost of illness Impact on quality of life (eg, QALYs or DALYs) Time-series data needed for economic modeling

Abbreviations: CSO, civil society organization; DALY, disability-adjusted life-year; EPI, Expanded Programme of Immunisation; Gavi, Gavi, the Vaccine Alliance; GNN, Global NITAG Network; IA2030, Immunization Agenda 2030; NITAG, National Immunization Technical Advisory Group; NRA, National Regulator Agency; QALY, quality-adjusted life-year; RITAG, Regional Immunization Technical Advisory Group; SAGE, Strategic Advisory Group of Experts on Immunization; SDG, Sustainable Development Goal; WHO, World Health Organization.

### Advocacy

Creating partnerships with private- and public-sector stakeholders, communicating the evidence-based value of an intervention, and seeking political commitment are key advocacy objectives for successful implementation of immunization and other public health prevention programs [2, 11, 12]. For a vaccine to be prioritized for introduction, the need for vaccination—both globally and among populations where the vaccine is to be used—must be well recognized [13]. However, the exact data needs and how they are described may vary for different stakeholders. Public health authorities may be most interested in health benefits, reduction in health service utilization, and cost-effectiveness; political leaders may focus on avoidable deaths or returns on investments from a vaccination program; public and private donors may prioritize the impact of new vaccines (or treatments) arising from research; and the general public may respond most to individual stories and data relevant to vulnerable groups such as children or the elderly.

An effective advocacy tool enables comparisons between the burden of the targeted disease and other diseases already prioritized for vaccine introduction or diseases with high public awareness. Communicating burden of disease data within the context of global public health goals (eg, Sustainable Development Goals [SDGs], specifically SDG3 targeting good health and well-being through prevention of communicable diseases [1]) and the potential contribution of interventions such as vaccination in achieving these goals, is important to capture the attention of global and national decision-makers. Ideally, burden of disease data should encompass the complete disease spectrum of a pathogen to accurately convey its importance, while also focusing on clinical manifestations (and accompanying data) of the greatest public health significance. Where country-specific estimates of disease burden are lacking, it is important to provide regional estimates to assist with decision making [14].

### Regulatory Oversight and Licensure

Vaccine licensure requires well-designed vaccine efficacy studies (or studies of accepted correlates of protection such as immunogenicity) that measure the impact of the vaccine candidate on prevention of well-defined, pathogen-specific disease endpoints of clinically significant severity, as well as a careful assessment of vaccine-associated adverse events. Contemporary local disease data are needed to identify clinical trial sites for conducting such efficacy studies. Age-specific incidence estimates of the clinical disease endpoint that the vaccine is targeting are needed to design adequately powered trials. These disease endpoints, key clinical indications, and target populations are often defined in WHO Preferred Product Characteristics. Background rates of disease-related conditions using standard burden of disease measures from populations where vaccine trials are being conducted can provide context for evaluations of potential of vaccine-associated disease [15]. Data describing the natural progression of disease from acute infection to associated sequelae or chronic disease as well as safety data are important considerations for this objective. Key stakeholders include vaccine developers and manufacturers, clinical trial sponsors, and regulatory authorities, as well as global, regional, and national vaccine policy-making bodies (Table 1). To facilitate swift and successful implementation, vaccine developers and those synthesizing burden of disease data should have a good understanding of the needs for regulatory approval and for funding decisions.

### Policy and Post-Licensure Evaluation

Burden of disease estimates are among the key considerations for policy decisions on vaccine introduction. These estimates, along with cost-effectiveness data, enable comparison with other public health priorities and facilitate prioritization of a vaccine for inclusion in national programs. Population-level socioeconomic indicators (eg, income level, access to healthcare, water, and sanitation) and environmental factors that influence disease burden are also important considerations in making equity-based vaccination policies and maximizing their impact [16].

In addition, high-quality surveillance is essential to fill data gaps, enhance the credibility of the burden estimates, and provide epidemiological data needed to optimize the use of preventive strategies. The establishment of surveillance systems using standardized methods is essential for measurements of “real world” vaccine effectiveness and impact, and for monitoring epidemiological trends (eg, changes in peak age of infection or in serotype or genotype prevalence of the pathogen) post-implementation. These assessments may be useful for justifying the continued use of the vaccine and informing vaccination policies and schedules. For certain vaccines (eg, pneumococcal conjugate vaccine), WHO recommends at least 2 years of pre-vaccine data and 3–5 years of post-vaccine data to appropriately make such decisions [17].

In addition to measuring the performance of a vaccine, vaccine efficacy trials can be used to estimate vaccine-preventable disease burden by measuring the proportion of non-specific clinical syndromes prevented by the vaccine [18]. Such clinical syndromes of public health importance that can be caused by multiple pathogens include pneumonia, diarrhea, and meningitis. Data requirements for this objective should therefore include pathogen-specific and non-specific disease endpoints and, where feasible, be population-based. This differs from data required to measure efficacy against a pathogen-specific disease endpoint observed in phase III clinical trials conducted in the setting of idealized standard-of-care and Good Clinical Practice.

The key stakeholders for the policy objective are the immunization technical advisory groups at global, regional, and national levels and global bodies, like WHO, which many resource-poor countries look to for advice and guidance.

### Financing

The financing objective refers to the post-licensure financing of vaccine introduction and scale-up, as opposed to financing pre-licensure vaccine research. A key global stakeholder is Gavi, the Vaccine Alliance, which supports the introduction of new vaccines in the poorest countries. Its vaccine investment strategy analyses provide ranking criteria for the evaluation of vaccines that includes the economic impact (direct and indirect costs averted) and health impact (cases and deaths averted) [19]. However, national governments are primarily responsible for long-term sustainable financing of their immunization programs. High-quality disease data facilitate economic analyses, including cost-effectiveness, benefit–cost, and societal return on investment analyses of vaccination. Burden of disease data on, and economic analysis of, all clinical endpoints are important as the drivers of a vaccine’s value may be from endpoints that are of lesser severity but may contribute significantly to population-level healthcare costs compared with those outcomes requiring high individual medical care but a low population incidence. The full societal value of a vaccine, which may include the value from changes in educational attainment, labor force participation and productivity, antimicrobial resistance (AMR) levels, public health surveillance expenditure, and social equity, should also be considered, and has become important for COVID-19 vaccines [6].

## APPLICATIONS TO STREP A VACCINE DEVELOPMENT

**Figure 1.**
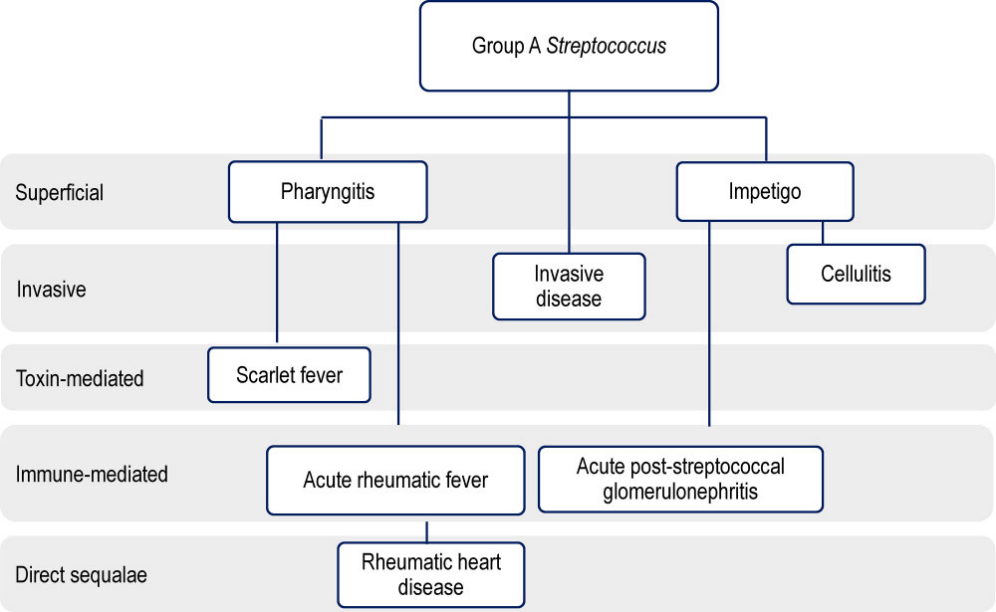
Key group A *Streptococcus* disease syndromes. Colonization of upper respiratory infection and skin is not included. Note this is a simplified figure of the diseases associated with group A *Streptococcus.* Locally invasive disease and invasive disease can also include bacteremia, meningitis, puerperal sepsis, and necrotizing fasciitis. Toxin-mediated diseases can also include streptococcal toxic shock syndrome. Direct sequelae can also include chronic kidney disease. Figure adapted with permission by Cannon et al [20].

The clinical spectrum of Strep A is broad (Figure 1). The lack of a single, focused disease entity likely contributes to the lack of consensus on its public health priority and affects advocacy efforts for disease prevention through development of a Strep A vaccine. Additionally, while some clinical endpoints are specific to Strep A, others (eg, cellulitis, pharyngitis) have multiple etiologies.

To address this challenge and highlight the need for a Strep A vaccine, we used our framework to provide a roadmap and identify priority data requirements specific to key Strep A clinical endpoints, grouped into acute diseases (Table 2) and immune-mediated sequelae (Table 3). Some data purposes differ between high-income countries (HICs) and LMICs. However, the HIC/LMIC dichotomy does not account for the large heterogeneity within some HICs. For example, Australia has the highest reported rates of impetigo [21] and rheumatic heart disease (RHD) [22] in the world among First Nations people; hence, burden of disease data requirements in these populations in Australia may reflect more of an LMIC prioritization than an HIC. Furthermore, the simple dichotomy does not account for the transition from low-income to middle-income country status, which is particularly important for Gavi funding considerations and future vaccine implementation [23].

**Table 2.** Priorities for Data Requirements Describing Burden of Disease Across Vaccine Development and Evaluation Objectives for Acute Group A *Streptococcus* Diseases

Clinical Endpoint	Vaccine Objective			
	Advocacy	Regulatory/Licensure	Policy and Post-Licensure Evaluation	Financing
Pharyngitis (children)	Passive or active surveillance data measuring age-specific disease incidence and strain (eg, *emm* type) distribution Data on population transmission Vaccine acceptance HICs: Markers of immune response to differentiate asymptomatic carriage vs acute infection LMICs: Syndromic surveillance data to establish need for subnational vs regional estimates for countries lacking capacity; Strep A–specific pharyngitis data where feasible	Prospective, active surveillance with laboratory-confirmed clinical endpoints Strain-specific disease incidence where possible HICs: Establish infrastructure and data mechanisms for phase II/III vaccine clinical trials Monitor adverse events/safety from vaccine candidates Markers of immune response to assess asymptomatic carriage vs acute infection LMICs: Correlate with pre-existing syndromic surveillance sites	Prospective and retrospective data measuring age-specific (or reporting age-standardized) incidence rates (pre- and post-vaccine introduction) Trends in antibiotic use (and AMR in Strep A and bystander pathogens) over time HICs: Economic value of vaccine Estimates of herd immunity LMICs: Correlate with pre-existing syndromic surveillance sites Strep A–specific in limited sentinel sites	Retrospective economic (cost of illness) data from all available levels of health service indicators, but primarily general practice HICs: Level and cost of antibiotic use plus trends in AMR Economic value of vaccine
Impetigo (children)	Passive or active surveillance data measuring age-specific disease incidence and prevalence Vaccine acceptance HICs: Strep A–specific (laboratory-confirmed) where possible Data from a limited number of sentinel settings are adequate (as impetigo unlikely to be major driver in HICs) LMICs: Syndromic surveillance data with laboratory confirmation from selected high-performing sites	Prospective active surveillance with laboratory-confirmed clinical endpoints HICs: Only required in a small number of sentinel sites Phase II/III vaccine clinical trials unlikely to be feasible (given low disease prevalence) LMICs: Measure disease incidence/prevalence from selected regional sites Identify sites with adequate resources for future vaccine trials	Prospective and retrospective data measuring age-specific or age-standardized incidence/prevalence rates (pre- and post-vaccine introduction) Does not need to be Strep A–specific HICs: Potential basis for later vaccine effectiveness evaluation LMICs: Strep A–specific in a subset of sentinel sites	Retrospective economic (cost of illness) data from all available levels of health service indicators
Cellulitis	Prospective and retrospective passive and active surveillance data measuring age-specific disease incidence and prevalence HICs: Measure disease outcomes Strep A–specific data from limited sites if feasible	Not critical as initial efficacy needs to be demonstrated for pharyngitis and impetigo HICs: Consider phase III trials in targeted populations (eg, recurrent cellulitis in diabetics or elderly)	Incidence/prevalence rates, focusing on adults Does not need to be Strep A–specific LMICs: Syndromic surveillance data may be useful to monitor temporal trends	Retrospective economic (cost of illness) data from all available levels of health service indicators HICs: Lost productivity data Measure severe disease outcomes LMICs: Unlikely to be a priority
Invasive Strep A	Prospective and retrospective passive and active surveillance data measuring age-specific disease incidence and outcomes, including mortality Societal/economic burden HICs: Serotype (eg, *emm* type) data important High-risk populations (eg, First Nations) as likely to influence decision making LMICs: Establish sentinel site surveillance in geographically representative areas	Not critical as initial efficacy needs to be demonstrated for pharyngitis and impetigo but need to plan for post-licensure evaluation HICs: Strain-specific endpoints useful for post-licensure evaluations in some countries	Prospective and retrospective data measuring age-specific or age-standardized incidence/prevalence rates (age group will depend on clinical focus) HICs: Laboratory-confirmed infections Assess some key foci separately (eg, puerperal sepsis) Impact on AMR of group A strep LMICs: Strain-specific data from several select, high-performing sites	Retrospective economic (cost of illness) data focusing on hospitalizations and mortality Data on imputations and other sequelae, including DALYs where possible
Scarlet fever	Prospective and retrospective passive and active surveillance data measuring age-specific disease incidence	Not critical as initial efficacy needs to be demonstrated for pharyngitis and impetigo but may be observable in some settings	Prospective and retrospective data measuring age-specific or age-standardized incidence rates *HIC:* Serotype data important Trends in antibiotic use (and AMR) over time	Retrospective economic (cost of illness) data from, primarily, general practice HICs: Level and cost of antibiotic use plus trends in AMR

Abbreviations: AMR, antimicrobial resistance; DALY, disability-adjusted life-year; HIC, high-income country; LMIC, low- and middle-income country; Strep A, group A *Streptococcus*.

**Table 3.** Priorities for Data Requirements Describing Burden of Disease Across Vaccine Development and Evaluation Objectives for Immune-Mediated Sequalae of Group A *Streptococcus*

	Objective			
Clinical Endpoint	Advocacy	Regulatory/Licensure	Policy and Post-Licensure Evaluation	Financing
ARF	Age-specific incidence and changes over time HICs: Unlikely to be a driver except in First Nation sub-populations	Not critical as initial efficacy needs to be demonstrated for pharyngitis and impetigo but need to plan for post-licensure evaluation HICs: Relevant for First Nation sub-populations LMICs: Determination of pathway for evaluating impact on severe disease outcomes from early acute infection	Age-specific incidence and changes over time. Attack rates from acute diseases to ARF (difficult to obtain) HICs: Relevant for First Nation sub-populations LMICs: Data on socioeconomic indicators Progression from acute infection	Retrospective economic (cost of illness) data targeted to hospitalizations and treatment HICs: Relevant for First Nation sub-populations
RHD	Prevalence in certain at-risk groups HICs: Relevant for First Nation sub-populations LMICs: Severity of RHD	Not critical as initial efficacy needs to be demonstrated for pharyngitis and impetigo but need to plan for post-licensure evaluation LMICs: Determination of pathway for evaluating impact on severe disease outcomes from early acute infection	Age-specific prevalence and temporal changes Need to understand progression from acute infection (pharyngitis) to estimate long-term reduction from pharyngitis prevention HICs: Relevant for First Nation sub-populations LMICs: Data on socioeconomic indicators Progression from acute infection	Retrospective economic (cost of illness) data targeted to hospitalizations, treatment, and mortality HICs: Relevant for First Nation sub-populations
APSGN	Not a critical driver for advocacy	Not required as efficacy needs to be demonstrated for pharyngitis	Plan for post-licensure evaluation of impact	Retrospective economic (cost of illness) data targeted to hospitalizations and treatment LMICs: Potential impact on long-term chronic renal disease

Abbreviations: APSGN, acute post-streptococcal glomerulonephritis; ARF, acute rheumatic fever; HIC, high-income country; LMIC, low- and middle-income country; RHD, rheumatic heart disease.

In application of this framework to Strep A, we have considered the target population, established by the WHO Preferred Product Characteristics [24], to be infants and/or young children. Pharyngitis and impetigo have been strategically targeted as initial, feasible clinical endpoints of a Strep A vaccine [24]. Both are associated with considerable health burden and are assumed to be primary intermediates on the causal pathway to immune-mediated diseases, such as acute rheumatic fever (ARF) and RHD, and a proportion of invasive Strep A diseases. While these efficacy targets have been set irrespective of the strain and serotype coverage of a future Strep A vaccine, we consider the importance of strain-specific data for certain vaccine objectives—in particular, advocacy and regulatory oversight and licensure. This is particularly important as the *emm* types of Strep A causing disease vary across geographical settings and over time [25]. While some vaccine candidates are not serotype specific, varying multivalent vaccines are also in development [26] and serotype replacement may be an important consideration when collating or evaluating data on Strep A burden of disease.

### Acute Diseases

Prioritization of disease burden estimates of acute Strep A clinical syndromes varies by vaccine objective. For advocacy, it is important to focus on clinical endpoints that are most likely to influence decision making, such as invasive Strep A, which is the highest driver of overall Strep A deaths in most HICs. The incidence of invasive Strep A has remained high or is increasing in multiple countries (eg, the United States [27], United Kingdom [28], and Australia [29]) but likely to be underrecognized in LMICs, potentially hampering advocacy requirements. Scarlet fever has also recently increased across the United Kingdom [30]. Data to support vaccine acceptability are also critical to support advocacy efforts, given that vaccine hesitancy is a well-recognized global issue [31].

For the regulatory/licensure objective, burden of disease data need to be pathogen- and, where possible, strain-specific. Active prospective surveillance with laboratory-confirmed clinical endpoints and focused on the early vaccine development targets (pharyngitis and impetigo) will be necessary to generate burden of disease data for this objective. Sites with established active surveillance for pharyngitis or impetigo can transition to become phase II or III vaccine trial sites.

Policy review often assesses the impact of vaccination on all-cause disease syndromes (eg, all-cause pharyngitis, cellulitis, pneumonia, or sepsis). Due to the mild–moderate symptomatology of Strep A pharyngitis, estimates of disease incidence should include community surveillance data and data from primary care, healthcare clinics, and emergency departments to enable policy decisions within the health sector. While impetigo is generally seen as an issue requiring primary care and also requires community surveillance, a high hospitalization burden in First Nations populations (eg, Australia [32]) has been noted using International Classification of Diseases, 10th Revision (ICD-10), coded skin infections. These data will prove useful for post-licensure population studies evaluating disease trends pre- and post-vaccine introduction. Given increasing global concerns of AMR, the use of burden of disease data to measure vaccine impact on AMR and estimate reductions in antibiotic use is an additional important requirement for the policy objective [33].

Cost-of-illness data are essential for financing decisions. However, the drivers of costs differ across acute Strep A endpoints and different socioeconomic settings. For pharyngitis, the associated consequences of increasing AMR and parental productivity losses are likely key drivers for the value of vaccination; the importance of population AMR trends is recognized by WHO’s vaccine roadmap [24]. It was recently estimated that 17% of antibiotic prescriptions for pharyngitis among US children could be prevented by a Strep A vaccine [34]. For invasive Strep A, the drivers of burden are more likely to be hospitalizations and mortality; hence, data requirements need to be targeted to address these.

### Immune-Mediated Sequelae

Rheumatic heart disease has been a major focus of Strep A burden of disease research [35, 36] and more advocacy efforts, including the need for a safe and effective vaccine, are established (eg, the global call to action from the American Heart Association and the World Health Assembly [37]) compared with those for other Strep A endpoints. While initial efficacy of a Strep A vaccine is primarily focused on acute endpoints, data to describe the progression from acute and common conditions (eg, pharyngitis and impetigo) [38] to chronic conditions like ARF and RHD are needed, in particular for the policy and financing objectives. This will provide key stakeholders critical information necessary to alleviate any vaccine safety concerns and have sufficient confidence in modeling forecasts that estimate vaccine impact on severe outcomes.

For policy evaluation, longitudinal data describing the changes in incidence and prevalence will be needed pre- and post-vaccine introduction to assist in understanding population vaccine impact. Knowledge of background ARF and RHD rates in jurisdictions of high incidence is important, especially in light of concerns of enhanced disease following vaccination—one of the prior impediments to a Strep A vaccine development [39]. Additionally, for LMICs, data on country-level socioeconomic indicators are likely to be important determinants of population-level disease burden.

## IDENTIFICATION OF RESEARCH PRIORITIES

Guided by this framework, we have identified 4 research priorities for Strep A burden of disease.

1. *Establish sentinel surveillance sites for pharyngitis (and impetigo) measuring age-specific disease burden.* To address the regulatory and licensure objective, surveillance sites need to be established to facilitate future vaccine trials. Such activities are underway through the Australian Strep A Vaccine Initiative (ASAVI; www.asavi.org.au) and surveillance activities in remote Australia [40], but surveillance sites in LMICs are needed. For LMICs, adequate local surveillance infrastructure is necessary to build awareness of future benefits of a vaccine [3, 41].
2. *Collate data to describe the incidence of invasive Strep A in LMICs*. There is currently a dearth of published age-specific data in LMICs, which is critical for advocacy, policy evaluation, and financing vaccine objectives. Leveraging Strep A data from existing surveillance networks, focusing on LMICs, presents an option to fill this data gap.
3. *Assess the attributable fraction of Strep A to cellulitis*. Cellulitis has been demonstrated as a major contributor to Strep A burden, cost, and therefore value of a vaccine in Australia [20] and New Zealand [42]. The burden of Strep A cellulitis in other jurisdictions is unknown. Synthesis and analysis of existing data or designing prospective studies are critical to understanding age-specific rates for advocacy objectives. This will likely become increasingly important with future Strep A vaccines targeting adult populations [24].
4. *Develop Strep A burden of disease estimates through the Global Burden of Disease (GBD) Project*. There are few Strep A–specific burden estimates. These will become increasingly important, especially for advocacy, to compare its disease burden with that of other diseases within and across countries.

Other research priorities identified include the following: (1) the need to better understand the drivers of country, regional, and international vaccine decision making with increased connections through the relevant immunization technical advisory groups; (2) multi-country epidemiological record linkage studies using administrative health data across all Strep A endpoints; and (3) quantifying antibiotic use for pharyngitis.

## CONCLUSIONS

We developed a framework that describes the different requirements and components of burden of disease data to address 4 interrelated but distinct vaccine objectives—advocacy, regulatory oversight and licensure, policy, and financing. By using these 4 different lenses, we described the data needs across the spectrum of Strep A clinical outcomes and identified research priorities that map to and will facilitate achieving each of these critical vaccine objectives. This framework is meant to be dynamic, being both updated as Strep A vaccine candidates progress through clinical trials and development, as well as adapted for other diseases with defined vaccine needs. Importantly, its use is envisaged in coordinating the vaccine research and development efforts among international stakeholders and setting the IA2030 research and innovation agenda for Strep A and other important diseases on the vaccine development horizon.

## Supplementary Material

ciac291_Supplementary_Data

## Supplementary Data

Supplementary materials are available at *Clinical Infectious Diseases* online. Consisting of data provided by the authors to benefit the reader, the posted materials are not copyedited and are the sole responsibility of the authors, so questions or comments should be addressed to the corresponding author.

## Notes

***Author Contributions.*** H. C. M., J. W. C., D. C. K., J. C., and C. V. B. developed the framework through consultation with the SAVAC Burden of Disease Working Group (BoDWG). The BoDWG includes 13 members from 7 geographically diverse countries (representing all except for 1 of the 6 WHO regions), with expertise covering Strep A and other VPDs, disease surveillance, and vaccine program implementation. Further details on the BoDWG can be found at https://savac.ivi.int/what_we_do. The BoDWG operates on a purely volunteer basis with no operational budget and meets virtually. All members agreed on the 4 vaccine objectives and were invited to provide detailed comments on the structure and content of the framework. H. C. M. drafted the first version of the manuscript. All other co-authors provided input into the framework. All named authors reviewed the manuscript and provided feedback and edits. All authors approved the final manuscript and the decision to submit for publication.

***Acknowledgments.*** The authors thank Jean-Louis Excler for his helpful comments on the framework.

***Financial support.*** This work was supported by the International Vaccine Institute, the Wellcome Trust (grant number 215490/Z/19/Z), and the National Health and Medical Research Council of Australia (to A. C. B. and J. W. C.).

***Members of the SAVAC Burden of Disease Working Group.*** Jonathan Carapetis, Chris Van Beneden, David C. Kaslow, Thomas Cherian, Theresa Lamagni, Mark Engel, Jeffrey W. Cannon, Hannah C. Moore, Asha C. Bowen, Anna Seale, Gagandeep Kang, David Watkins and Sam Kariuki.
