# Supplementary material for: A Systematic Framework for Prioritizing Burden of Disease Data Required for Vaccine Development and Implementation: The Case for Group A Streptococcal Diseases
Source: Clin Infect Dis. 2022 Apr 19;75(7):1245–54. doi: 10.1093/cid/ciac291 (PMC9525082; doi:10.1093/cid/ciac291)
Supplement: ciac291_Supplementary_Data [file ciac291_supplementary_data.docx]

# Supplementary Material

Supplementary Table 1

**Examples of successful vaccination programs across a variety of infectious diseases aligned with the four different vaccine objectives**

| **Vaccine Objective** | **Examples** |
| --- | --- |
| **Advocacy** | *Pneumococcal conjugate vaccines (PCV):*   - Development of “World Pneumonia Day” ([www.stoppneumonia.org](http://www.stoppneumonia.org)) to raise awareness of pneumococcal disease as an important cause of pneumonia and the value of PCV vaccination - PneumoADIP (Pneumococcal Vaccines Accelerated Development and Introduction Plan, [www.preventpneumo.org](http://www.preventpneumo.org)) initiative was established to accelerate the introduction of pneumococcal vaccines in LMIC[1] - The All Party Parliamentary Group (APPG) for Pneumonia and Pneumococcal Diseases was established in the United Kingdom and advocated for pneumonia prevention through pneumococcal vaccination[2] |
|  | Haemophilus influenzae *type b (Hib) conjugate vaccines:*   - The Hib Initiative promoted global evidence-based communication and advocacy through the collation and dissemination of data[3] which led to the introduction of Hib vaccines in Gavi-eligible countries[4] |
|  | *Meningococcal vaccines:*   - The MenAFriNet international consortium ([www.menafrinet.org](http://www.menafrinet.org)) established a regional surveillance network and led advocacy efforts to inform vaccine development and implementation of Meningitis A vaccine programs for the meningitis belt in sub-Saharan Africa[5] |
| **Regulatory / Licensure** |  |
|  | *COVID-19 vaccines:*   - Recommended use of COVID-19 vaccines for children aged 12-17 years in the US following careful review of vaccine efficacy data, monitoring of vaccine adverse events among COVID-19 vaccine recipients, and epidemiologic data among children with COVID-19 [6]. This US recommendation was since updated to include children aged 5 years and over (www.cdc.gov/coronavirus/2019-ncov/vaccines/recommendations/children-teens.html) |
|  | *Respiratory syncytial virus (RSV) vaccines:*   - Reports of enhanced disease following use of a formalin-inactivated RSV vaccine[7] halted further vaccine development for decades |
|  | *PCV:*   - Establishment of regulatory guidance for the production and quality control of pneumococcal conjugate vaccines, including the serological criteria for licensure of new products facilitated the licensure of new PCV products[8] |
| **Policy and Post-Licensure Evaluation** | *PCV:*   - Continual assessment of disease burden and epidemiology (including serotype replacement) through established post-licensure evaluation provided evidence for changing immunisation schedules and vaccine formulations[9] - Use of established surveillance mechanisms to measure disease burden prior to vaccine introduction and use of non-specific disease endpoints (e.g., pneumonia, otitis media) to measure PCV performance in addition to more specific endpoint of invasive pneumococcal disease[10, 11] |
|  | *Seasonal influenza:*   - Nationwide surveillance programs of hospitalised influenza and measurement of vaccine effectiveness led to the introduction of nationally-funded preschool vaccination programs[12] |
| **Financing** | *PCV:*   - Cost-effectiveness studies using US surveillance data for a range of clinical endpoints to quantify the indirect effects and therefore the cost-effective use of health care resources[13] |
|  | *COVID-19:*   - Widespread societal costs and economic loses from COVID-19 pandemic resulted in expedited vaccine development[14] |
|  | *Measles:*   - Evidence of high return on investment for measles vaccination[15] used to justify widespread inclusion of measles vaccines in national immunisation programs |

## References

1. Levine OS, Cherian T, Shah R, Batson A. PneumoADIP: an example of translational research to accelerate pneumococcal vaccination in developing countries. J Health Popul Nutr **2004**:268-274.

2. Turner D, Taylor R. Parliament unites to tackle global problem. BMJ **2008**;336:233-234.

3. Hajjeh R. Accelerating introduction of new vaccines: barriers to introduction and lessons learned from the recent Haemophilus influenzae type B vaccine experience. Philos Trans Royal Soc Lond B Biol Sci **2011**;366:2827-2832.

4. Ojo LR, O’Loughlin RE, Cohen AL, et al. Global use of Haemophilus influenzae type b conjugate vaccine. Vaccine **2010**;28:7117-7122.

5. Novak RT, Moïsi JC, Tall H, et al. Country Data for Action: The MenAfriNet Experience in Strengthening Meningitis Surveillance in Africa. J Infect Dis **2019**;220:S137-S139.

6. Gargano J, Wallace M, Hadler S, et al. Use of mRNA COVID-19 vaccine after reports of myocarditis among vaccine recipients: update from the Advisory Committee on Immunization Practices—United States, June 2021. Morbidity and Mortality Weekly Report (MMWR) **2021**;70:977-982.

7. Kapikian AZ, Mitchell RH, Chanock RM, Shvedoff RA, Stewart CE. An epidemiologic study of altered clinical reactivity to respiratory syncytial (RS) virus infection in children previously vaccinated with an inactivated RS virus vaccine. Am J Epidemiol **1969**;89:405-421.

8. Jódar L, Butler J, Carlone G, et al. Serological criteria for evaluation and licensure of new pneumococcal conjugate vaccine formulations for use in infants. Vaccine **2003**;21:3265-3272.

9. Varghese L, Talbot L, Govender A, Zhang X-H, Mungall BA. A cost-effectiveness analysis of the 10-valent pneumococcal non-typeable Haemophilus influenzae protein D conjugate vaccine (PHiD-CV) compared to the 13-valent pneumococcal conjugate vaccine (PCV13) for Universal Mass Vaccination Implementation in New Zealand. Appl Health Econ HealthPpolicy **2018**;16:331-345.

10. Fathima P, Gidding HF, McIntyre PB, et al. Effectiveness of pneumococcal conjugate vaccine against hospital admissions for pneumonia in Australian children: a retrospective, population-based, record-linked cohort study. Lancet Child Adoles Health **2019**;3:713-724.

11. Wasserman M, Chapman R, Lapidot R, et al. Twenty-Year Public Health Impact of 7-and 13-Valent Pneumococcal Conjugate Vaccines in US Children. Emerg Infect Dis **2021**;27:1627.

12. Blyth CC, Macartney KK, McRae J, et al. Influenza epidemiology, vaccine coverage and vaccine effectiveness in children admitted to sentinel Australian hospitals in 2017: results from the PAEDS-FluCAN collaboration. Clin Infect Dis **2019**;68:940-948.

13. Ray GT, Pelton SI, Klugman KP, Strutton DR, Moore MR. Cost-effectiveness of pneumococcal conjugate vaccine: an update after 7 years of use in the United States. Vaccine **2009**;27:6483-6494.

14. Bloom DE, Cadarette D, Ferranna M. The Societal Value of Vaccination in the Age of COVID-19. Am J Public Health **2021**;111:1049-1054.

15. Sim SY, Watts E, Constenla D, Brenzel L, Patenaude BN. Return On Investment From Immunization Against 10 Pathogens In 94 Low-And Middle-Income Countries, 2011–30: Study estimates return on investment from immunization programs against ten pathogens for ninety-four low-and middle-income countries from 2011 to 2030. Health Affairs **2020**;39:1343-1353.
